# Efficacy, immunogenicity and safety of the AS04‐HPV‐16/18 vaccine in Chinese women aged 18‐25 years: End‐of‐study results from a phase II/III, randomised, controlled trial

**DOI:** 10.1002/cam4.2399

**Published:** 2019-07-15

**Authors:** Feng‐Cai Zhu, Shang‐Ying Hu, Ying Hong, Yue‐Mei Hu, Xun Zhang, Yi‐Ju Zhang, Qin‐Jing Pan, Wen‐Hua Zhang, Fang‐Hui Zhao, Cheng‐Fu Zhang, Xiaoping Yang, Jia‐Xi Yu, Jiahong Zhu, Yejiang Zhu, Feng Chen, Qian Zhang, Hong Wang, Changrong Wang, Jun Bi, Shiyin Xue, Lingling Shen, Yan‐Shu Zhang, Yunkun He, Haiwen Tang, Naveen Karkada, Pemmaraju Suryakiran, Dan Bi, Frank Struyf

**Affiliations:** ^1^ Jiangsu Province Center for Disease Prevention and Control Nanjing China; ^2^ National Cancer Center ‐ Cancer Hospital Chinese Academy of Medical Sciences (CAMS) & Peking Union Medical College (PUMC) Beijing China; ^3^ Affiliated Drum Tower Hospital of Nanjing University Medical School Nanjing China; ^4^ Lianshui Center for Disease Prevention and Control Lianshui China; ^5^ Jintan Center for Disease Prevention and Control Jintan China; ^6^ Xuzhou Center for Disease Prevention and Control Xuzhou China; ^7^ Binhai Center for Disease Prevention and Control Yancheng China; ^8^ GSK Shanghai China; ^9^ GSK Bangalore India; ^10^ GSK Wavre Belgium; ^11^ Present address: Leo‐Pharma R&D China Hub Shanghai China

**Keywords:** AS04‐HPV‐16/18 vaccine, China, efficacy, human papillomavirus, immunogenicity, safety

## Abstract

**Background:**

Cervical cancer is a major public health concern in China. We report the end‐of‐study results of a phase II/III trial to assess the efficacy, immunogenicity, and safety of the AS04‐human papillomavirus (HPV)‐16/18 vaccine in Chinese women aged 18‐25 years followed for up to 72 months after first vaccination. Results of approximately 57 months following first vaccination have been previously reported.

**Methods:**

Healthy 18‐25‐year‐old women (N = 6051) were randomized (1:1) to receive three doses of AS04‐HPV‐16/18 vaccine or Al(OH)_3_ (control) at Months 0‐1‐6. Vaccine efficacy against HPV‐16/18 infection and cervical intraepithelial neoplasia (CIN), cross‐protective vaccine efficacy against infections and lesions associated with nonvaccine oncogenic HPV types, immunogenicity, and safety were assessed. Efficacy was assessed in the according‐to‐protocol efficacy (ATP‐E) cohort (vaccine N = 2888; control N = 2892), total vaccinated cohort for efficacy (TVC‐E; vaccine N = 2987; control N = 2985) and TVC‐naïve (vaccine N = 1660; control N = 1587).

**Results:**

In initially HPV‐16/18 seronegative/DNA‐negative women, vaccine efficacy against HPV‐16/18‐associated CIN grade 2 or worse was 87.3% (95% CI: 5.5, 99.7) in the ATP‐E, 88.7% (95% CI: 18.5, 99.7) in the TVC‐E, and 100% (95% CI: 17.9, 100) in the TVC‐naïve. Cross‐protective efficacy against incident infection with HPV‐31, HPV‐33 and HPV‐45 was 59.6% (95% CI: 39.4, 73.5), 42.7% (95% CI: 15.6, 61.6), and 54.8% (95% CI: 19.3, 75.6), respectively (ATP‐E). At Month 72, >95% of initially seronegative women who received HPV vaccine in the ATP cohort for immunogenicity (N = 664) remained seropositive for anti‐HPV‐16/18 antibodies; anti‐HPV‐16 and anti‐HPV‐18 geometric mean titers were 678.1 EU/mL (95% CI: 552.9, 831.5) and 343.7 EU/mL (95% CI: 291.9, 404.8), respectively. Serious adverse events were infrequent (1.9% vaccine group [N = 3026]; 2.7% control group [N = 3025]). Three and zero women died in the control group and the vaccine group respectively. New onset autoimmune disease was reported in two women in the vaccine group and two in the control group.

**Conclusions:**

This is the first large‐scale randomized clinical trial of HPV vaccination in China. High and sustained vaccine efficacy against HPV‐16/18‐associated infection and cervical lesions was demonstrated up to Month 72. The vaccine had an acceptable safety profile. Combined with screening, prophylactic HPV vaccination could potentially reduce the high burden of HPV infection and cervical cancer in China.

**Trial registration:**

NCT00779766.

## Graphical abstract

Cervical cancer is one of the most aggressive forms of cancer that women can suffer from. The presence of human papillomavirus (HPV) is responsible for almost all cases of cervical cancer. For these reasons, the world health organization recommends that HPV vaccines should be included in national vaccination programs. From the first licensure of the first HPV vaccine in 2006 to the development of new HPV vaccines, the latter have been studied in clinical trials and demonstrated to decrease in the number of cervical cancers while remaining acceptably safe. Many developed countries have included HPV vaccination in their program. These countries reported a decrease of cervical cancer cases. Many countries from the less developed regions are still yet to introduce HPV vaccination. The novelty: In 2016, the Chinese authorities approved licensure of the first HPV vaccine for use in China to help prevent cervical cancer and based their decision on clinical trials conducted in China. We present here the final results of the key clinical trial of this HPV vaccine that involved around 6000 Chinese women over 6 years. The impact: Chinese women represent almost 10% of the worldwide population at risk of cervical cancer. Latest estimations from 2016 indicate that 111 000 new cervical cancer cases are reported every year in China. This number could be reduced with the HPV vaccination.

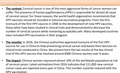

## 1 INTRODUCTION

Persistent infection with oncogenic human papillomavirus (HPV) types has been recognized as an essential cause of cervical cancer and precancer.^1^, ^2^, ^3^ Cervical cancer is a major public health concern in China, with almost 100 000 cases and over 30 000 deaths estimated in 2015.^4^ In women aged 15‐44 years, it is the second most common cancer and the third most common cause of cancer‐related death in China.^5^ Overall HPV prevalence in the general population is 17.7%, according to a pooled analysis of 17 population‐based studies,^6^ with the first peak of infection with oncogenic HPV types occurring in women 15‐19 years of age.^7^ Common with worldwide data, HPV‐16 and HPV‐18 are the most prevalent HPV types associated with cervical cancer in China.^5^

All sexually active women are at risk of HPV infection, and it is therefore important that prophylactic HPV vaccination programs target girls before they begin sexual activity. In a cross‐sectional epidemiological survey across urban and rural areas of China, there was a trend towards earlier sexual debut (median 17 years) and riskier sexual behaviors in younger cohorts of Chinese women.^8^ HPV vaccination for girls before they begin sexual activity would likely contribute to the prevention of HPV infection and associated disease in China, as has been demonstrated in other countries.^9^, ^10^, ^11^

The AS04‐HPV‐16/18 vaccine (*Cervarix*, GSK) is licensed in over 135 countries and was approved in China in July 2016 for use in females 9‐25 years of age. The licence was extended for use in females 9‐45 years of age in May 2018. The vaccine has been shown to offer protection against infection, cytological abnormalities and cervical intraepithelial neoplasia (CIN) associated with HPV‐16/18, including CIN grade 3 or worse, the closest surrogate endpoint to cervical cancer.^12^, ^13^, ^14^, ^15^, ^16^, ^17^, ^18^ Cross‐protection against nonvaccine types has also been reported.^14^, ^19^, ^20^, ^21^ Sustained antibody responses have been demonstrated almost 10 years postvaccination,^22^ and the vaccine has an acceptable safety profile.^12^, ^23^

Here we report the end‐of‐study analysis up to 72 months of follow‐up of a phase II/III trial in Chinese women aged 18‐25 years. The results of two previous event‐triggered analyses, with follow‐up up to 57 months following first vaccination, have been reported.^24^, ^25^ This is the first large RCT with the longest follow‐up of vaccine efficacy in the prevention of HPV infection and related cervical precancer in China. We also present data from exploratory analyses not previously reported evaluating efficacy of the vaccine against CIN irrespective of HPV type.

## 2 MATERIALS AND METHODS

### 2.1 Study design and participants

Details of the study design and participants have been published previously.^24^, ^26^ This randomized, controlled, double‐blind study evaluated the efficacy, safety and immunogenicity of the AS04‐HPV‐16/18 vaccine in healthy Chinese women aged 18‐25 years. The trial was conducted in accordance with the Declaration of Helsinki (1996) and the International Conference on Harmonisation Good Clinical Practice guidelines, and the protocol and informed consent form were approved by the ethics committees of the Center for Disease Control and Prevention (CDC) Jiangsu Province and the Cancer Foundation of China. All participants provided written informed consent. The study is registered with http://ClinicalTrials.gov, number NCT00779766.

Women were enrolled at four sites in Jiangsu province. All women living in the areas covered by the study sites who met the eligibility criteria were invited to participate. Women who were pregnant or breastfeeding, or had chronic or autoimmune disease under treatment or immunodeficiency were excluded. Full inclusion and exclusion criteria have been described previously.^24^, ^26^ The vaccine or control was administered in a three‐dose schedule (0, 1 and 6 months). The initial study was scheduled for 24 months, with an optional extension to 48 months and a second optional extension to 72 months. Study visits were scheduled for each participant at Months 0, 1, 6, 7, 12, 18 and 24. Visits at Months 30, 36, 42 and 48 were scheduled for women who took part in the first extension. Women who took part in the second extension had 2 or 3 further visits, depending on the time of their enrolment, at Months 60, 66 and 72.

Women were randomized 1:1 to receive either the AS04‐HPV‐16/18 vaccine or control (aluminium hydroxide), which were supplied in identical prefilled syringes. A randomization list was generated by the study sponsor using a standard Statistical Analysis System (SAS) program. A randomization blocking scheme was used to ensure balance between treatments. Treatment allocation was performed at the investigator's site using a central randomization system on internet (SBIR). The randomization algorithm used a minimization process accounting for study center. Participants, investigators, and study staff were blinded to treatment allocation and HPV DNA and serology results throughout the whole study period. The study blinding was maintained until database freeze for this end‐of‐study analysis.

### 2.2 Procedures

Cervical samples were obtained every 6 months for HPV DNA and cytology testing (according to the protocol‐defined algorithm). A polymerase chain reaction (PCR) assay using specific SPF_10_ primers amplifying a 65‐nucleotide region of the HPV L1 gene was used to test cervical samples and biopsy material for HPV DNA from most known HPV types. HPV‐positive specimens were typed by reverse hybridization line probe assay (LiPA) using 28 HPV‐specific hybridization probes, which allowed detection of 14 oncogenic and 11 nononcogenic HPV types. All HPV‐positive samples were also tested using HPV‐16 specific and HPV‐18 specific PCR.^27^

Cervical cytology was performed using the ThinPrep Pap Test (Cytec Corporation, Boxborough, USA) and samples were evaluated according to the Bethesda 2001 classification system. CIN terminology was used for reporting of histologically defined cervical lesions.^28^ Histopathology was handled centrally. Biopsy and excisional treatment specimens were reviewed by a panel of expert pathologists. Agreement from at least two panel members was required on the location and grade of the lesion. Final CIN case assignments were reviewed and agreed by an independent endpoint committee.

Antibody responses against HPV‐16 and HPV‐18 were determined using enzyme‐linked immunosorbent assay (ELISA)^29^ in the immunogenicity subset at Months 0, 7, 12, 24, 36, 48 and 72. Seropositivity was defined as an antibody titer greater than the assay cut‐off value. While monitoring the quality of the assay, a high variability was observed in the low range of assay results from unvaccinated trial participants.^30^ Therefore from Month 36, the assay cut‐off value was changed from 8 EL.U/mL to 19 EL.U/mL for HPV‐16 and from 7 EL.U/mL to 18 EL.U/mL for HPV‐18.

Serious adverse events (SAEs), new onset chronic diseases (NOCD), new onset autoimmune diseases (NOAD), medically significant conditions, pregnancy and pregnancy outcomes were recorded throughout follow‐up. Medically significant conditions were defined as: adverse events (AEs) prompting emergency room or physician visits that were not (1) related to common diseases or (2) routine visits for physical examination or vaccination, or SAEs that were not related to common diseases. Common diseases included: upper respiratory infections, sinusitis, pharyngitis, gastroenteritis, urinary tract infections, cervicovaginal yeast infections, menstrual cycle abnormalities and injury.

### 2.3 Outcomes

The primary endpoint was vaccine efficacy against a combined endpoint of 6‐month persistent infection (6MPI) with HPV‐16 and/or HPV‐18 (HPV‐16/18) and/or CIN grade 1 or worse (CIN1+) associated with HPV‐16/18. Secondary endpoints were incident infection, 6MPI, 12‐month persistent infection (12MPI), atypical squamous cells of undetermined significance or worse (ASC‐US+), CIN1+, and CIN grade 2 or worse (CIN2+), associated with HPV‐16/18 or with nonvaccine oncogenic HPV types individually or in combination. 6MPI was defined as at least two positive HPV DNA PCR assays for the same viral genotype with no negative DNA sample between, over an interval of approximately 6 months; 12MPI was defined in the same way over an interval of approximately 12 months. CIN1+ was defined as CIN1, CIN2, CIN3, adenocarcinoma in situ (AIS) or invasive cervical cancer. CIN2+ excluded CIN1. Vaccine efficacy irrespective of HPV DNA in the lesion was evaluated as an exploratory endpoint in the end‐of‐study analysis. Immunogenicity and safety endpoints were followed throughout the entire study period.

### 2.4 Statistical analysis

We have previously published confirmatory event‐driven analyses reporting efficacy against 6MPI/CIN1+ (primary endpoint)^24^ and against CIN2+ (secondary endpoint).^25^ The end‐of‐study analysis reported here was descriptive and was intended to confirm and expand the efficacy results of the previous event‐driven analyses. The main focus of the present analysis was on vaccine efficacy against CIN2+. The sample size calculation was done for the confirmatory analyses on 6MPI/CIN1+ and CIN2+, and has been reported previously.^24^, ^25^ Efficacy results are presented for a combined analysis of the initial study and the two optional extension studies. Thus, data from women who did not continue to the first or second extension study are nevertheless included in the end‐of‐study analysis up to the point at which they withdrew.

The according‐to‐protocol cohort for efficacy (ATP‐E) was the primary efficacy analysis cohort, comprising women with available efficacy data, who met all eligibility criteria, received three doses of vaccine or control, complied with the protocol and had normal or low‐grade cytology (ASC‐US or low‐grade squamous intraepithelial lesions) at baseline. Efficacy analyses were also conducted in the total vaccinated cohort for efficacy (TVC‐E), comprising women with available efficacy data, who received at least one dose of vaccine or control and had normal or low‐grade cytology at baseline, and in the TVC‐naïve, comprising women with available efficacy data, who received at least one dose of vaccine or control and at baseline were HPV DNA‐negative for all 14 oncogenic types tested, seronegative for HPV‐16 and HPV‐18 and had negative cytology. The primary analysis in the ATP‐E was performed in women DNA‐negative at baseline and Month 6 and seronegative at baseline for the HPV type considered in the analysis. In the TVC‐E, the analysis was conducted in women DNA‐negative and seronegative at baseline for the type considered in the analysis. Analyses in the ATP‐E and TVC‐E were also conducted in women DNA‐negative at baseline regardless of serostatus. All women in the TVC‐naïve were DNA‐negative for all oncogenic HPV types tested and seronegative at baseline for HPV‐16 and HPV‐18.

Vaccine efficacy was calculated using a conditional exact method, which computes an exact confidence interval (CI) around the rate ratio (ratio of event rates in the vaccine vs control groups), taking into account follow‐up time within each group. Vaccine efficacy was defined as 1 minus the rate ratio. In the previously reported confirmatory analyses, statistically significant vaccine efficacy was defined as the lower limit of the 95% CI above zero.^24^, ^25^ As mentioned already, the present end‐of‐study analysis was descriptive. Follow‐up in the ATP‐E cohort started on the day after the third vaccine dose, and in the TVC‐E and TVC‐naïve on the day after the first dose, and ended for each participant at the time of the event or, if no event was reported, at the time of last data available.

Immunogenicity was primarily analyzed in the ATP cohort for immunogenicity which included women in the immunogenicity subset with available immunogenicity data, who met all eligibility criteria, received three doses of vaccine or control and complied with the protocol. Women who acquired HPV‐16 or HPV‐18 infection during the study were excluded from the ATP cohort. Seropositivity rates with exact 95% CIs and geometric mean titers (GMTs) with 95% CIs were calculated for HPV‐16 and HPV‐18. GMTs were calculated by taking the antilog of the mean of the log titer transformations. Antibody levels below the assay cut‐off value were assigned an arbitrary value of half the cut‐off for the purpose of the calculation of GMT. Safety was analyzed in the total vaccinated cohort (TVC) which included all women who received at least one dose of vaccine or control. The percentage of participants with an adverse event was calculated with exact 95% CIs. SAS version 9.2 was used for all statistical analysis.

## 3 RESULTS

### 3.1 Study participants

**Figure 1:**
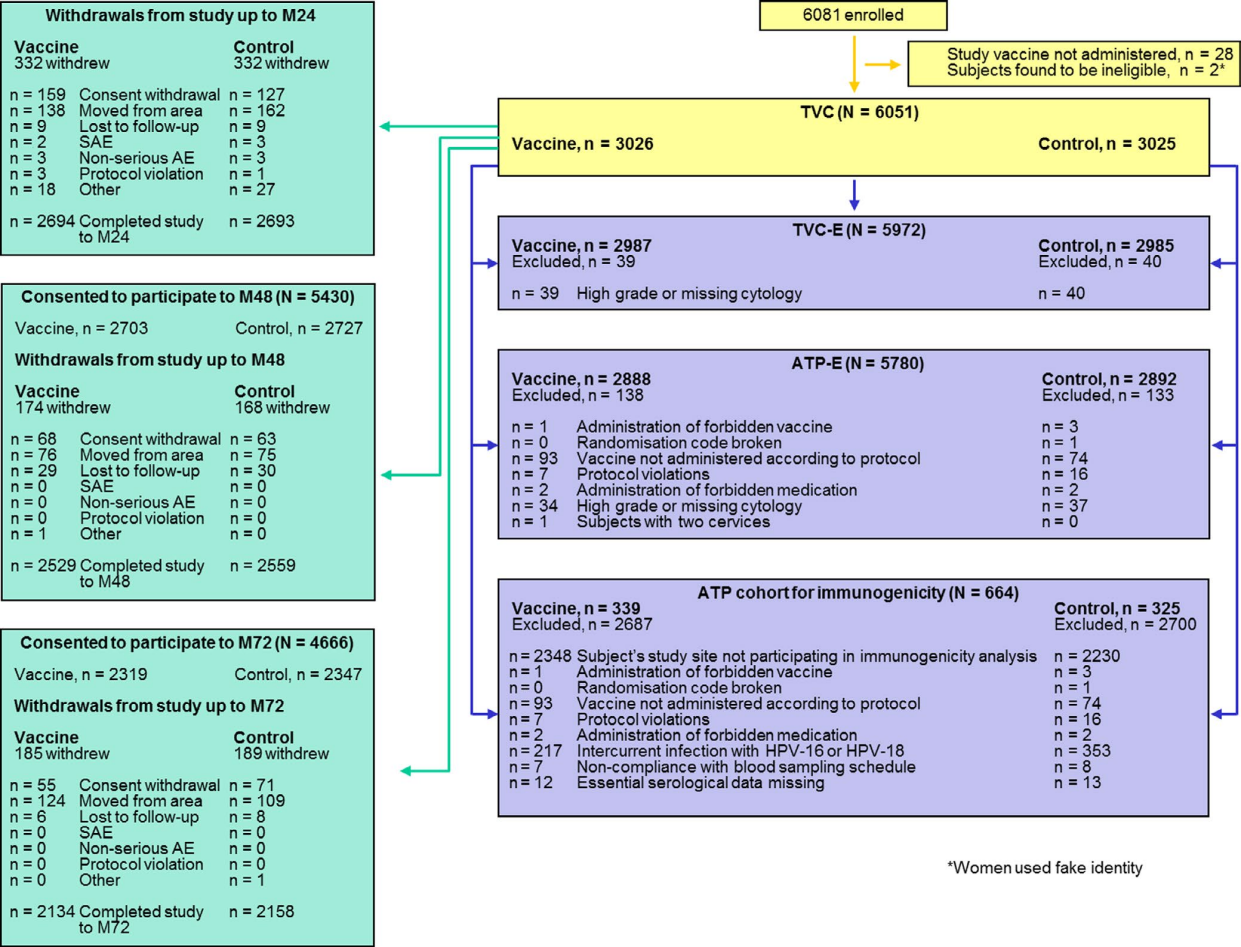
Participant disposition. The TVC‐naïve included 1660 women in the vaccine group and 1587 women in the control group. AE, adverse event; ATP‐E, according to protocol cohort for efficacy; N, number of women in the analysis; n, number of cases; SAE, serious adverse event; TVC‐E, total vaccinated cohort for efficacy; TVC‐naïve, total vaccinated naïve cohort; TVC, total vaccinated cohort

The first woman was enrolled in October 2008. Final data were collected in February 2016. Of 6051 women enrolled in the initial study, 5430 consented to participate in the first extension up to month 48, and 4666 women consented to participate in the second extension up to month 72 (2319 vaccine, 2347 control) (Figure 1). For the combined end‐of‐study analysis of the initial and two extension studies reported here, the TVC included 3026 women in the vaccine group and 3025 women in the control group. The TVC‐E included 98.7% of participants, and the ATP‐E included 95.5%. Full participant disposition is shown in Figure 1. The mean follow‐up time after the first dose in the TVC was approximately 62 months. In the ATP‐E, follow‐up began after the third dose, with a mean of approximately 57 months. Mean age at vaccination was 23 years in both study groups; the median age was 23 years (range 18‐32) in the vaccine group and 23 years (range 18‐39) in the control group of the TVC cohort. All women were of Chinese heritage. Further details of baseline characteristics have been reported previously.^26^

### 3.2 Vaccine efficacy

#### 3.2.1 Vaccine efficacy against HPV‐16/18

In the ATP‐E cohort in women seronegative at baseline, two cases of the primary endpoint, 6MPI/CIN1+ associated with HPV‐16/18, were reported in the vaccine group and 69 cases in the control group, resulting in a vaccine efficacy of 97.1% (95% CI: 89.1, 99.7) (Table 1). Vaccine efficacy was 97.8% (95% CI: 91.9, 99.7) in the ATP‐E regardless of serostatus, 94.3% (95% CI: 86.1, 98.2) in the TVC‐E in women seronegative at baseline, 93.8% (95% CI: 86.9, 97.6) in the TVC‐E regardless of serostatus, and 96.5% (95% CI: 86.9, 99.6) in the TVC‐naïve (Table 1). High vaccine efficacy against the secondary endpoints, incident infection, 6MPI, 12MPI and ASC‐US+, was also observed (Table 1).

**Table 1:** Vaccine efficacy against endpoints associated with HPV‐16/18

	Vaccine		Control		Vaccine efficacy, % (95% CI)
	N	n	N	n	
ATP‐E, women HPV‐16/18^a^ DNA‐negative at baseline and Month 6, and seronegative for HPV‐16/18^a^ at baseline					
6MPI/CIN1+	2523	2	2534	69	97.1 (89.1, 99.7)
Incident infection	2524	34	2534	156	78.4 (68.5, 85.5)
6MPI	2483	2	2492	63	96.8 (88.0, 99.6)
12MPI	2428	1	2458	32	96.9 (81.2, 99.9)
ASC‐US+	2521	5	2534	60	91.6 (79.4, 97.4)
CIN1+	2523	1	2534	15	93.3 (56.2, 99.8)
CIN2+	2523	1	2534	8	87.3 (5.5, 99.7)
ATP‐E, women HPV‐16/18^a^ DNA‐negative at baseline and Month 6, regardless of initial serostatus					
6MPI/CIN1+	2804	2	2801	91	97.8 (91.9, 99.7)
Incident infection	2806	49	2801	204	76.5 (67.8, 83.2)
6MPI	2762	2	2748	83	97.6 (91.1, 99.7)
12MPI	2702	1	2709	38	97.4 (84.5, 99.9)
ASC‐US+	2802	12	2801	83	85.7 (73.6, 92.9)
CIN1+	2804	1	2801	18	94.4 (64.8, 99.9)
CIN2+	2804	1	2801	10	90.0 (29.5, 99.8)
TVC‐E, women DNA‐negative and seronegative for HPV‐16/18^a^ at baseline					
6MPI/CIN1+	2567	5	2587	87	94.3 (86.1, 98.2)
Incident infection	2610	49	2639	184	73.5 (63.6, 81.1)
6MPI	2551	4	2571	80	95.0 (86.7, 98.7)
12MPI	2518	3	2536	41	92.6 (76.9, 98.5)
ASC‐US+	2563	7	2585	71	90.1 (78.5, 96.2)
CIN1+	2567	2	2587	17	88.1 (49.7, 98.7)
CIN2+	2567	1	2587	9	88.7 (18.5, 99.7)
TVC‐E, women HPV‐16/18^a^ DNA‐negative at baseline, regardless of initial serostatus					
6MPI/CIN1+	2853	7	2857	112	93.8 (86.9, 97.6)
Incident infection	2900	71	2914	238	70.8 (61.8, 77.9)
6MPI	2835	5	2839	103	95.2 (88.5, 98.5)
12MPI	2801	4	2795	50	92.0 (78.3, 97.9)
ASC‐US+	2849	14	2855	97	85.7 (74.8, 92.4)
CIN1+	2853	3	2857	21	85.7 (52.0, 97.3)
CIN2+	2853	2	2857	12	83.3 (24.8, 98.2)
TVC‐naïve, women DNA‐negative for all oncogenic HPV types tested and seronegative for HPV‐16 and 18 at baseline					
6MPI/CIN1+	1591	2	1530	55	96.5 (86.9, 99.6)
Incident infection	1616	32	1558	114	73.5 (60.4, 82.7)
6MPI	1588	2	1527	52	96.3 (86.1, 99.6)
12MPI	1572	1	1507	29	96.7 (80.1, 99.9)
ASC‐US+	1591	5	1530	46	89.6 (73.9, 96.8)
CIN1+	1591	0	1530	10	100 (56.9, 100)
CIN2+	1591	0	1530	6	100 (17.9, 100)

Abbreviations: 6MPI, 6‐month persistent infection; 12MPI, 12‐month persistent infection; ASC‐US+, atypical squamous cells of undetermined significance or worse; ATP‐E, according to protocol cohort for efficacy; CI, confidence interval; CIN1+, cervical intraepithelial neoplasia grade 1 or worse; CIN2+, cervical intraepithelial neoplasia grade 2 or worse; HPV, human papillomavirus; N, number of women in analysis; n, number of cases; TVC‐E, total vaccinated cohort for efficacy; TVC‐naïve, total vaccinated naïve cohort.

a For the corresponding type considered in the analysis.

New HPV infections continued to accrue throughout the study. At the month 24 analysis (initial study) in the ATP‐E cohort seronegative at baseline for HPV‐16/18, one case of 6MPI/CIN1+ associated with HPV‐16/18 was detected among 2497 women in the vaccine group and 17 cases among 2502 women in the control group.^24^ This compares with two cases among 2523 women in the vaccine group and 69 cases among 2534 women in the control group at the end‐of‐study analysis (combined data from the initial study and the two extension studies). Corresponding values for incident infection were 15/2497 (vaccine), 49/2502 (control) at month 24,^24^ compared with 34/2524 (vaccine) and 156/2534 (control) at the end of study. Likewise for ASC‐US, values were 1/2494 (vaccine) and 16/2502 (control) at month 24,^24^ vs 5/2521 (vaccine) and 60/2534 (control) at the end of study.

**Figure 2:**
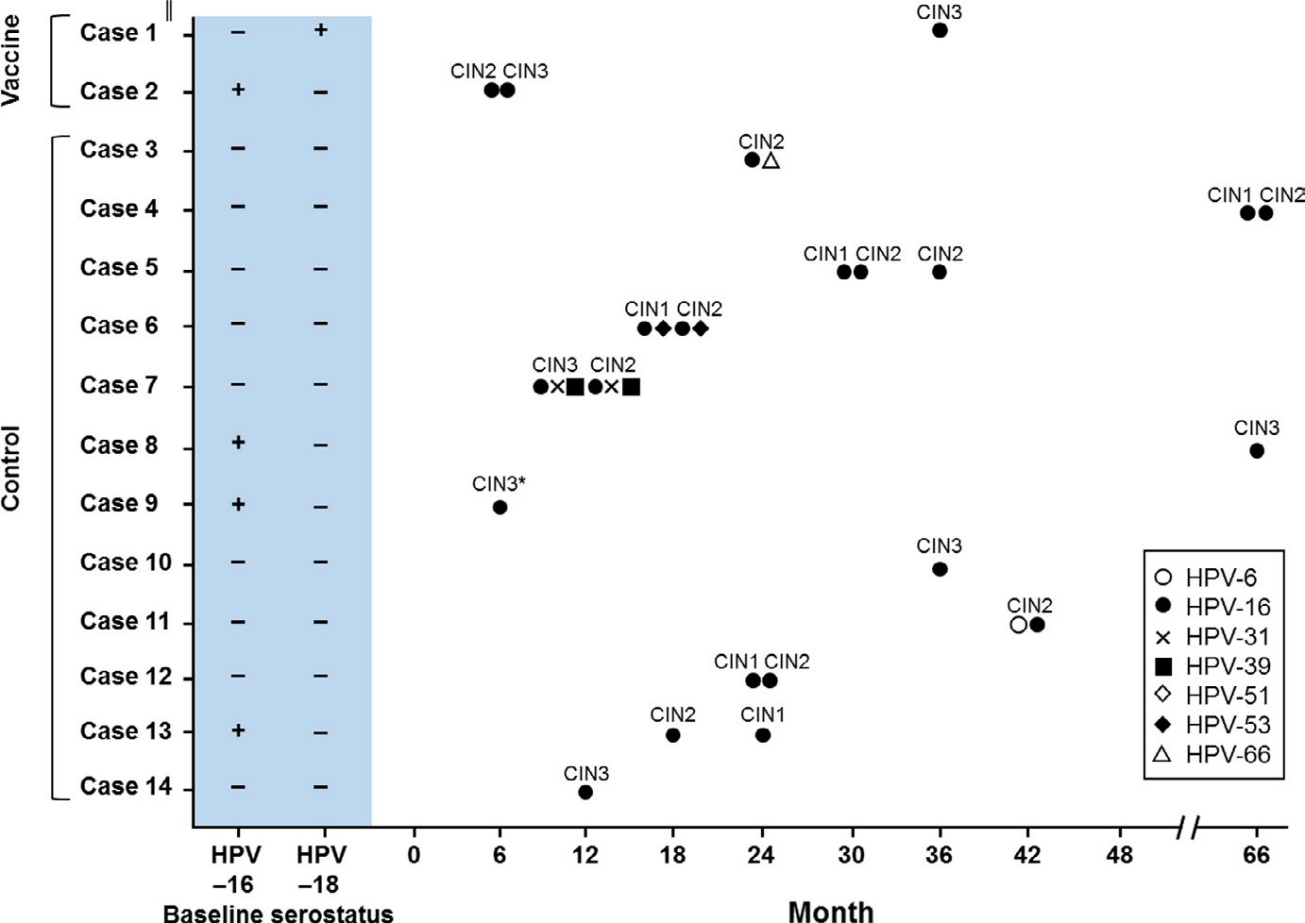
Clinical diagnosis and biopsy PCR results of CIN2+ cases associated with HPV‐16/18 (TVC‐E). Cases were from women in the TVC‐E who were HPV‐16/18 DNA‐negative at baseline regardless of initial serostatus. ‖Case 1 was also part of the ATP‐E cohort. *All samples taken by punch biopsy except case 9 which was taken by endocervical curettage. CIN1, cervical intraepithelial neoplasia grade 1; CIN2, cervical intraepithelial neoplasia grade 2; CIN3, cervical intraepithelial neoplasia grade 3; HPV, human papillomavirus; LSIL, low‐grade squamous intraepithelial lesion; PCR, polymerase chain reaction; TVC‐E, total vaccinated cohort for efficacy

A key focus of the end‐of‐study analysis was efficacy against CIN2+. Vaccine efficacy against CIN2+ associated with HPV‐16/18 was 87.3% (95% CI: 5.5, 99.7) in the ATP‐E in women DNA‐negative at months 0, 6 and seronegative at baseline for the corresponding type, 90.0% (95% CI: 29.5, 99.8) in the ATP‐E in women DNA‐negative regardless of initial serostatus, 88.7% (95% CI: 18.5, 99.7) in the TVC‐E in women DNA‐negative and seronegative for the corresponding type at baseline, 83.3% (95% CI: 24.8, 98.2) in the TVC‐E in women DNA‐negative at baseline regardless of initial serostatus and 100% (95% CI: 17.9, 100) in the TVC‐naïve in women DNA‐negative and seronegative for HPV‐16/18 at baseline (Table 1).Fourteen cases (two cases in the vaccine group and 12 in the control group) of CIN2+ associated with HPV‐16/18 were seen in the TVC‐E in women DNA‐negative at baseline regardless of serostatus (Figure 2).

The first CIN2+ case in the vaccine group (case 1) was a woman who was also included in the ATP‐E cohort. The woman was identified with a CIN3 associated with HPV‐16 at Month 36. At baseline, she was seropositive for HPV‐18 (but seronegative for HPV‐16, the type identified in the lesion) and had normal cytology. She had an infection with HPV‐16, HPV‐51 and HPV‐52 at Month 12, and had ASC‐US associated with HPV‐16 and HPV‐52 at Month 36. Assessment of causality was difficult due to multiple oncogenic HPV infections detected in both the lesion and the preceding cytological specimens. The second woman with CIN2+ in the vaccine group (case 2) was included in the TVC‐E (but not in the ATP‐E), and was seropositive for HPV‐16 and had ASC‐US associated with HPV‐52 at baseline. She was diagnosed with CIN3 associated with HPV‐16 at Month 6; she was also identified with infection with HPV‐33 at Months 6, 12 and 18. No new CIN2+ cases were reported between the previous event‐triggered analysis^25^ and this end‐of‐study analysis.

#### 3.2.2 Vaccine efficacy against nonvaccine types

The analysis was performed in women DNA‐negative at baseline for the HPV type considered in the analysis, regardless of initial HPV‐16/18 serostatus. Women in the ATP‐E analysis were also DNA‐negative at Month 6 for the HPV type considered. Vaccine efficacy against virological endpoints associated with nonvaccine HPV types in the ATP‐E and TVC‐E is shown in Table 2. In the ATP‐E cohort, vaccine efficacy was observed against incident infection with HPV‐31 (59.6% [95% CI: 39.4, 73.5]), HPV‐33 (42.7% [95% CI: 15.6, 61.6]) and HPV‐45 (54.8% [95% CI: 19.3, 75.6]), and against 6MPI and 12MPI associated with HPV‐31 (64.6% [95% CI: 27.6, 83.9] and 81.3% [95% CI: 34.5, 96.5], respectively) (Table 2). Similar values were observed in the TVC‐E cohort (Table 2).

**Table 2:** Vaccine efficacy against virological endpoints and ASC‐US+ associated with nonvaccine HPV types

	ATP‐E, women DNA‐negative for HPV type considered at baseline and Month 6, regardless of HPV‐16/18 serostatus					TVC‐E, women DNA‐negative for HPV type considered at baseline, regardless of HPV‐16/18 serostatus				
	Vaccine		Control		Vaccine efficacy, % (95% CI)	Vaccine		Control		Vaccine efficacy, % (95% CI)
	N	n	N	n		N	n	N	n	
Incident infection										
HPV‐31	2775	35	2769	86	59.6 (39.4, 73.5)	2875	44	2897	109	59.8 (42.4, 72.3)
HPV‐33	2768	43	2770	75	42.7 (15.6, 61.6)	2872	58	2897	94	38.2 (13.4, 56.2)
HPV‐35	2794	53	2790	65	18.2 (−19.5, 44.2)	2890	57	2908	77	25.6 (−6.2, 48.1)
HPV‐39	2747	154	2759	133	−17.1 (−48.8, 7.8)	2858	176	2888	164	‐8.5 (−35.0, 12.8)
HPV‐45	2782	18	2788	40	54.8 (19.3, 75.6)	2885	28	2908	53	46.8 (14.4, 67.6)
HPV‐51	2741	166	2742	204	18.5 (−0.5, 34.0)	2862	202	2867	227	10.7 (−8.4, 26.5)
HPV‐52	2655	251	2651	284	11.2 (−5.6, 25.4)	2798	315	2806	341	7.0 (−8.7, 20.5)
HPV‐56	2771	104	2766	113	7.9 (−21.3, 30.1)	2874	118	2887	129	8.3 (−18.7, 29.2)
HPV‐58	2760	83	2755	111	25.2 (−0.3, 44.4)	2865	96	2879	134	28.2 (6.0, 45.4)
HPV‐59	2802	52	2873	65	20.5 (−16.3, 45.9)	2898	57	2905	77	26.2 (−5.3, 48.5)
HPV‐66	2768	103	2751	124	17.0 (−8.6, 36.7)	2877	123	2877	145	15.1 (−8.7, 33.8)
HPV‐68	2763	101	2771	107	5.2 (−25.7, 28.5)	2875	124	2896	125	0.1 (−29.1, 22.7)
HPV‐31/33/45	2811	85	2810	175	52.0 (37.4, 63.4)	2904	113	2921	217	48.6 (35.1, 59.4)
HRW‐HPV	2811	744	2811	852	13.9 (4.9, 22.0)	2904	860	2921	965	11.6 (3.0, 19.5)
HR‐HPV	2811	764	2811	914	18.5 (10.2, 26.1)	2904	886	2921	1026	15.0 (7.0, 22.4)
6MPI										
HPV‐31	2734	11	2718	31	64.6 (27.6, 83.9)	2812	16	2824	41	60.9 (28.8, 79.5)
HPV‐33	2725	16	2716	30	46.6 (−1.2, 72.8)	2808	25	2823	37	31.9 (−16.2, 60.7)
HPV‐35	2750	19	2738	18	−5.9 (−113.9, 47.4)	2826	19	2834	24	20.4 (−51.5, 58.8)
HPV‐39	2703	63	2706	50	−27.3 (−88.4, 13.6)	2794	72	2816	63	−15.7 (−64.9, 18.7)
HPV‐45	2738	8	2737	10	19.5 (−126.6, 72.4)	2820	13	2834	17	23.0 (−68.5, 65.6)
HPV‐51	2702	69	2688	73	4.8 (−34.1, 32.5)	2799	84	2795	86	1.8 (−34.3, 28.2)
HPV‐52	2614	128	2606	117	−10.8 (−43.6, 14.4)	2737	165	2734	140	−19.6 (−50.9, 5.1)
HPV‐56	2728	43	2712	41	−5.0 (−65.2, 33.2)	2810	53	2815	46	−16.0 (−76.1, 23.3)
HPV‐58	2718	38	2703	49	22.4 (−21.0, 50.6)	2800	45	2807	60	24.8 (−12.6, 50.0)
HPV‐59	2758	16	2730	16	0.3 (−113.1, 53.3)	2833	20	2830	22	8.9 (−74.9, 52.9)
HPV‐66	2726	36	2697	37	3.0 (−57.8, 40.4)	2812	43	2804	41	−5.1 (−65.3, 33.1)
HPV‐68	2723	32	2718	36	10.7 (−47.9, 46.3)	2810	45	2823	45	‐0.7 (−55.8, 34.9)
HPV‐31/33/45	2767	33	2756	68	51.6 (25.6, 69.1)	2839	52	2846	90	42.3 (17.9, 59.8)
HRW‐HPV	2767	381	2757	418	8.6 (−5.2, 20.7)	2839	466	2846	487	3.5 (−9.9, 15.2)
HR‐HPV	2767	382	2757	466	18.7 (6.7, 29.1)	2839	468	2846	542	13.9 (2.4, 24.1)
12MPI										
HPV‐31	2674	3	2679	16	81.3 (34.5, 96.5)	2779	3	2779	20	85.0 (49.3, 97.1)
HPV‐33	2666	9	2678	9	‐0.5 (−185.8, 64.7)	2775	13	2778	11	‐19.2 (−193.8, 50.7)
HPV‐35	2689	12	2698	9	‐33.9 (−259.8, 48.2)	2792	12	2790	14	13.8 (−100.7, 63.6)
HPV‐39	2643	25	2667	22	‐14.8 (−113.5, 37.9)	2760	32	2772	28	‐15.7 (−99.4, 32.5)
HPV‐45	2677	5	2697	2	‐152.1 (−2547.2, 58.7)	2786	7	2791	6	‐17.6 (−323.5, 66.2)
HPV‐51	2642	28	2650	25	‐12.9 (−101.8, 36.6)	2767	38	2750	32	‐19.4 (−97.3, 27.4)
HPV‐52	2555	72	2571	65	‐11.9 (−59.0, 21.1)	2704	98	2694	76	‐30.5 (−78.4, 4.3)
HPV‐56	2667	14	2675	12	‐17.1 (−177.2, 49.7)	2776	16	2770	14	‐15.0 (−154.3, 47.4)
HPV‐58	2659	15	2664	18	16.5 (−75.6, 60.8)	2768	19	2764	22	13.2 (−68.0, 55.6)
HPV‐59	2697	8	2690	2	‐299.8 (−3764.5, 20.2)	2799	10	2785	4	‐150.8 (−995.3, 27.7)
HPV‐66	2665	17	2660	10	‐70.0 (−315.6, 26.5)	2779	22	2759	13	‐69.6 (−266.4, 18.3)
HPV‐68	2662	12	2678	12	‐0.7 (−144.9, 58.6)	2778	18	2779	19	4.7 (−91.8, 52.8)
HPV‐31/33/45	2706	16	2716	27	40.6 (−14.3, 70.1)	2805	22	2801	37	40.4 (−3.7, 66.5)
HRW‐HPV	2706	196	2717	192	‐3.0 (−26.4, 16.0)	2805	253	2801	239	‐7.3 (−28.6, 10.4)
HR‐HPV	2706	196	2717	219	10.3 (−9.3, 26.4)	2805	254	2801	273	6.4 (−11.4, 21.4)
ASC‐US+										
HPV‐31	2771	12	2769	32	62.5 (25.2, 82.4)	2826	17	2839	40	57.3 (22.9, 77.3)
HPV‐33	2764	17	2770	32	46.7 (1.1, 72.2)	2821	23	2838	37	37.3 (−8.4, 64.4)
HPV‐35	2790	20	2790	21	4.4 (−85.4, 50.8)	2839	20	2850	24	16.1 (−58.5, 56)
HPV‐39	2743	49	2759	35	‐41.6 (−125.1, 10.1)	2807	56	2831	43	‐32.1 (−101.3, 12.8)
HPV‐45	2778	9	2788	12	24.4 (−95.4, 71.9)	2834	11	2850	15	26.0 (−72.4, 69.3)
HPV‐51	2738	64	2742	78	17.5 (−16.3, 41.7)	2812	75	2810	87	13.5 (−19.2, 37.4)
HPV‐52	2651	83	2651	81	‐3.7 (−42.6, 24.6)	2749	99	2749	95	‐5 (−40.7, 21.5)
HPV‐56	2767	46	2766	51	9.6 (−37.4, 40.6)	2823	55	2829	55	‐0.7 (−49.1, 32)
HPV‐58	2756	36	2755	49	26.2 (−15.8, 53.4)	2814	42	2821	57	25.8 (−12.5, 51.4)
HPV‐59	2798	20	2783	17	‐17.7 (−139.2, 41.5)	2847	24	2846	23	‐4.7 (−94.2, 43.4)
HPV‐66	2764	42	2751	46	8.6 (−42, 41.3)	2826	48	2818	52	7.4 (−39.8, 38.8)
HPV‐68	2759	30	2771	37	18.4 (−35.8, 51.3)	2824	36	2838	43	15.6 (−34.6, 47.3)
HPV‐31/33/45	2807	34	2810	66	48.4 (20.9, 67.0)	2853	46	2862	81	43.1 (17.2, 61.2)
HRW‐HPV	2807	309	2811	352	12.0 (−2.8, 24.7)	2853	354	2862	394	9.7 (−4.5, 22.0)
HR‐HPV	2807	313	2811	386	19.2 (6.0, 30.6)	2853	357	2862	431	17.3 (4.6, 28.3)

Note

For combined types, women were DNA‐negative at baseline (ATP‐E and TVC‐E) and Month 6 (ATP‐E) for at least one of the HPV types considered in the analysis. Women could be infected with multiple HPV types. Therefore, the number of cases for combined types (HPV‐31/33/45, HRW‐HPV and HR‐HPV) might not equal the sum of the cases for each individual type included in the composite.

Abbreviation: 6MPI, 6‐month persistent infection; 12MPI, 12‐month persistent infection; ASC‐US+, atypical squamous cells of undetermined significance or worse; ATP‐E, according to protocol cohort for efficacy; CI, confidence interval; HPV, human papillomavirus; HR‐HPV, any oncogenic HPV type including HPV‐16 or HPV‐18; HRW‐HPV, any oncogenic HPV type except HPV‐16 and HPV‐18; N, number of women in analysis; n, number of cases; TVC‐E, total vaccinated cohort for efficacy.

In addition, vaccine efficacy was observed against ASC‐US+ associated with HPV‐31 (62.5% [95% CI: 25.2, 82.4]) and HPV‐33 (46.7% [95% CI: 1.1, 72.2]) in the ATP‐E; efficacy was similar in the TVC‐E but the lower limit of the 95% CI was below zero for HPV‐33 (Table 2). Vaccine efficacy in the ATP‐E against HPV‐31/33/45‐associated CIN1+ was 36.1% (95% CI: −80.5, 79.0), with seven cases in the vaccine group and 11 cases in the control group (Supporting information Table S1); vaccine efficacy against HPV‐31/33/45‐associated CIN2+ was 74.9% (95% CI: −25.8, 97.4), with three cases in the vaccine group and 18 cases in the control group (Supporting information Table S1). Number of cases of CIN2+ associated with HPV‐31 occurred in the vaccine group compared with four cases in the control group (vaccine efficacy: 100% [95% CI: −51.9, 100]) (Supporting information Table S1).

#### 3.2.3 Vaccine efficacy irrespective of HPV DNA in the lesion

**Figure 3:**
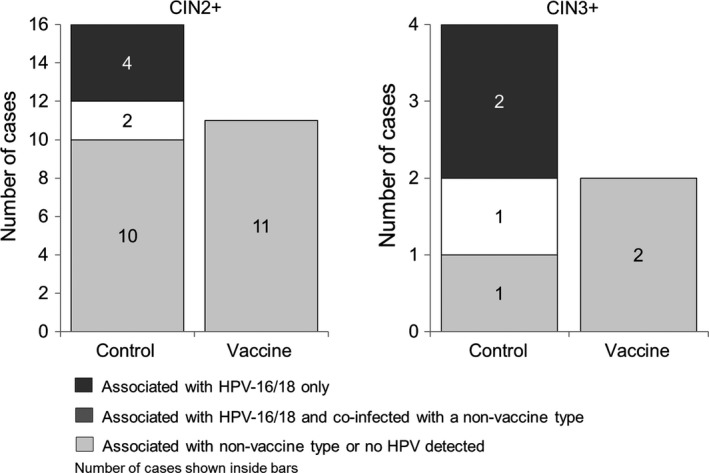
Number of cases of CIN2+ and CIN3+ associated with vaccine and nonvaccine types (TVC‐naïve). The incidence rate of CIN2+ irrespective of HPV was 0.13 per 100 person‐years in the vaccine group and 0.20 per 100 person‐years in the control group. The incidence rate of CIN3+ irrespective of HPV was 0.02 per 100 person‐years in the vaccine group and 0.05 per 100 person‐years in the control group. Oncogenic HPV types detected in CIN2+ cases in the vaccine group: no HPV detected (n = 2); HPV‐33 (n = 2); HPV‐35 (n = 1); HPV‐39 (n = 2); HPV‐51 (n = 1); HPV‐52/58 (n = 1); HPV‐58 (n = 1); HPV‐58/66 (n = 1). Oncogenic HPV types detected in CIN2+ cases in the control group: HPV‐16 (n = 4); HPV‐16/31/39 (n = 1); HPV‐16/66 (n = 1); HPV‐33 (n = 3); HPV‐35 (n = 1); HPV‐35/52 (n = 1); HPV‐45 (n = 1); HPV‐52 (n = 1); HPV‐58 (n = 3). Oncogenic HPV types detected in CIN3+ cases in the vaccine group: HPV‐58 (n = 1); HPV‐39 (n = 1). Oncogenic HPV types detected in CIN3+ cases in the control group: HPV‐16 (n = 2); HPV‐16/31/39 (n = 1); HPV‐58 (n = 1) CIN2+: cervical intraepithelial neoplasia grade 2 or worse. CIN3+, cervical intraepithelial neoplasia grade 3 or worse; HPV, human papillomavirus; TVC‐naïve, total vaccinated naïve cohort

Vaccine efficacy against CIN2+ irrespective of HPV DNA in the lesion was assessed in the TVC‐naïve. A total of 27 CIN2+ cases was observed at the end of the study, 11 in the vaccine group and 16 in the control group, resulting in a vaccine efficacy point estimate of 33.5% (95% CI: −52.6, 72.1). None of the cases in the vaccine group was associated with HPV‐16/18 (Figure 3). The most frequent nonvaccine types associated with CIN2+ in the vaccine group were HPV‐58, −33, −39, −35, and −52 (Figure 3). Six cases of CIN3+ were observed, two in the vaccine group and four in the control group (Figure 3). HPV‐39 and −58 were associated with the two cases of CIN3+ observed in the vaccine group.

### 3.3 Immunogenicity

**Figure 4:**
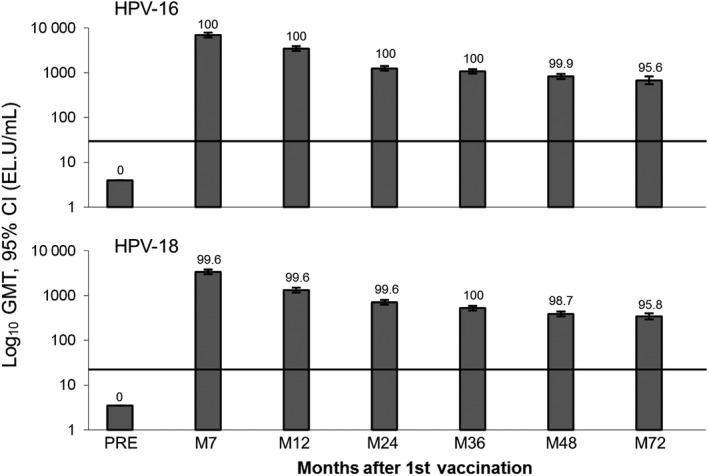
Immunogenicity in initially seronegative women receiving the HPV‐16/18 vaccine (ATP cohort for immunogenicity). Numbers above bars show the percentage seropositivity. Solid line shows the level of antibodies observed following clearance of a natural infection ie GMTs observed in women who were HPV‐16/18 DNA‐negative and seropositive at baseline in a phase III efficacy study (29.8 EL.U/mL for HPV‐16 and 22.6 EL.U/mL for HPV‐18).^34^ ATP, according to protocol; CI, confidence interval; EL.U, ELISA units; GMT, geometric mean titer; HPV, human papillomavirus; M, month; Pre, prevaccination

Immunogenicity against HPV‐16 and HPV‐18 was sustained throughout the study (Figure 4). Seropositivity at Month 72 was >95% for both HPV‐16 and HPV‐18 antibodies in initially seronegative women who received the HPV vaccine. GMTs were 678.1 EL.U/mL (95% CI: 552.9, 831.5) for HPV‐16 and 343.7 EL.U/mL (95% CI: 291.9, 404.8) for HPV‐18 at Month 72.

### 3.4 Safety

Reactogenicity and adverse events following AS04‐HPV‐16/18 vaccine administration in this study have been reported previously.^24^ Safety and pregnancy outcomes are shown in Table 3. SAEs were reported in 56 women in the vaccine group and in 81 women in the control group; the most common event was appendicitis, reported in four women in the vaccine group and 12 in the control group. One SAE was considered by the investigator to be possibly related to vaccination: a gastrointestinal tract reaction following vaccination in a woman receiving control vaccine. Three women in the control group died, one with a recorded cause of gastric neoplasm and two through suicide. No deaths were considered related to vaccination. No women in the vaccine group died. A medically significant condition was reported in 186 (6.1%) and 185 (6.1%) women in the vaccine and control groups, respectively. NOCDs and NOADs were rare and none was considered related to vaccination (Table 3).

**Table 3:** Safety and pregnancy outcomes throughout study (TVC)

		No. (%) women reporting outcome		
		Vaccine N = 3026		Control N = 3025
Safety outcomes				
Serious adverse event^a^		56 (1.9)		81 (2.7)
Medically significant condition^b^		186 (6.1)		185 (6.1)
New onset chronic disease^c^, ^d^		9 (0.3)		12 (0.4)
Hyperthyroidism		0		1 (0.0)
Allergy to arthropod bite		0		1 (0.0)
Anaphylactic reaction		1 (0.0)		0
Hypersensitivity		1 (0.0)		1 (0.0)
Arthritis		0		1 (0.0)
Gestational diabetes		0		1 (0.0)
Dermatitis allergic		3 (0.1)		4 (0.1)
Dermatitis atopic		1 (0.0)		0
Dermatitis contact		0		1 (0.0)
Psoriasis		1 (0.0)		1 (0.0)
Urticaria		2 (0.1)		2 (0.1)
New onset autoimmune disease^c^		2 (0.1)		2 (0.1)
Hyperthyroidism		0		1 (0.0)
VIIth nerve paralysis		1 (0.0)		0
Psoriasis		1 (0.0)		1 (0.0)
Deaths		0		3
Pregnancy outcomes				
Number of pregnancies			837	853
Live infant with no congenital abnormality			648 (77.4)	657 (77.0)
Live infant with congenital anomaly^e^			5 (0.6)	0
Spontaneous abortion^f^			16 (1.9)	13 (1.5)
Elective termination, no congenital anomaly			139 (16.6)	158 (18.5)
Elective termination, with congenital anomaly^g^			1 (0.1)	2 (0.2)
Ectopic pregnancy			7 (0.8)	7 (0.8)
Stillbirth, no congenital anomaly			0	2 (0.2)
Stillbirth, with congenital anomaly^h^			0	1 (0.1)
Lost to follow‐up			20 (2.4)	13 (1.5)

Abbreviations: IDMC, Independent Data Monitoring Committee; N, number of women; NOAD, new onset autoimmune disease; NOCD, new onset chronic disease; TVC, total vaccinated cohort.

a Serious adverse events were defined as any untoward medical occurrence that results in death, is life‐threatening, requires hospitalization or prolongs hospitalization, results in disability or incapacity, or congenital anomaly or birth defect in the offspring of a study subject.

b Medically significant conditions were defined as adverse events prompting either emergency room visits, physician visits that are not routine or related to common diseases, or serious adverse events that are not related to common diseases.

c A predefined list of potential NOCDs was reviewed by the IDMC. Based on this prespecified list, the clinical database was searched for all potential NOCDs and reviewed in a blind manner by a GlaxoSmithKline physician prior to data analysis. An event was considered to be a potential NOCD if it had not been recorded in the previous medical history of the woman (ie new onset) and/or if symptoms were characteristic of an NOCD. A separate list, restricted to potential autoimmune events, was also reviewed by the IDMC and was used by the GlaxoSmithKline safety physician to identify NOADs.

d One woman in the control group experienced two symptoms: allergy to arthropod bite and dermatitis allergic.

e Congenital anomalies were defined as structural‐morphological, chromosomal and genetic anomalies. Anomalies observed were cleft lip and palate (two infants), muscular torticollis (two infants) and foramen ovale patent (one infant).

f No congenital anomalies were identified among the spontaneous abortions.

g Down's Syndrome (vaccine group), hydrocephalus and achondroplasia (control group).

h Papyraceous fetus.

A total of 837 and 853 pregnancies were recorded in the vaccine and control groups, respectively (Table 3). Most pregnancies (77%) resulted in live infants with no congenital anomalies; 18% of women had an elective termination. Three infants were stillborn, all in the control group. Nine congenital anomalies were reported, five in liveborn infants in the vaccine group (two with a cleft lip and palate, two with muscular torticollis and one with foramen ovale patent), one in an elective termination in the vaccine group (Down's Syndrome), two in elective terminations in the control group (hydrocephalus and achondroplasia), and one in a stillborn infant in the control group (papyraceous fetus) (Table 3). No pattern in the nature of congenital anomalies was observed and none was considered possibly related to vaccination by the investigator. Sixteen (1.9%) and 13 (1.5%) women in the vaccine and control groups, respectively, experienced a spontaneous abortion, none of which was associated with a congenital anomaly (Table 3).

## 4 DISCUSSION

Data from this study have been previously described.^24^, ^25^ Here, we report data at the end of the study with up to 6 years of follow‐up, the longest follow‐up of the efficacy of an HPV vaccine in China. We have shown high, sustained vaccine efficacy against HPV‐16/18‐associated persistent infection and cervical lesions, and noteworthy evidence of cross‐protection with the AS04‐HPV‐16/18 vaccine.

CIN2+ is the traditional surrogate efficacy endpoint used in HPV vaccine trials. Indeed, the follow‐up time of the current study was extended to 72 months after the first vaccine dose to allow further evaluation of vaccine efficacy for this endpoint. At the end of the study, a high vaccine efficacy against CIN2+ associated with HPV‐16/18 was observed in all study cohorts, with 100% vaccine efficacy in the TVC‐naïve cohort. Vaccine efficacy does not appear to be impacted in a major way by previously cleared HPV infection in the ATP cohort (ie in women regardless of initial serostatus vs seronegative for HPV‐16/18 at baseline); this has been previously shown in women who were HPV‐16/18 seropositive before vaccination.^31^

In the two CIN2+ cases associated with HPV‐16 in the vaccine group of the TVC‐E, infections with HPV‐16, HPV‐51 and HPV‐52 were detected in one woman, and with HPV‐16 and HPV‐33 in the other woman. Although both cases were assigned as CIN2+ associated with HPV‐16 according to the HPV type assignment algorithm,^32^ definite causality could not be established. The phenomenon of coinfection with multiple oncogenic HPV types and the observed vaccine efficacy are in line with previous studies of the AS04‐HPV‐16/18 vaccine conducted in similar age groups in a wide range of countries.^12^, ^13^, ^14^, ^15^ Data from the large PATRICIA study suggest that efficacy estimates increase as time progresses^12^, ^32^, ^33^; this is likely to be a result of continued accrual of cases in the control group, with few cases observed over time in the vaccine group. This observation is supported by data from our study which indicate that efficacy of the AS04‐HPV‐16/18 vaccine is sustained in Chinese women up to 72 months of follow‐up. Sustained vaccine efficacy has been previously reported up to nearly 10 years post vaccination in other populations.^22^

Evaluation of cross‐protective efficacy against nonvaccine HPV types is a challenge in HPV vaccine trials.^19^ Because nonvaccine types are less common than HPV‐16 or HPV‐18, large sample size and lengthy follow‐up are usually required in order to accrue sufficient endpoint cases for statistically meaningful analysis. Most vaccine trials, including the present study, are not statistically powered to evaluate cross‐protection. Nevertheless, the study showed evidence of vaccine efficacy against incident infection with HPV‐31, HPV‐33 and HPV‐45, and against 6MPI and 12MPI with HPV‐31. These data are generally consistent with previous reports of efficacy against HPV‐31, HPV‐33 and HPV‐45.^13^, ^14^, ^16^, ^21^ However, the findings must be viewed with caution, given the caveats mentioned above and the limitations of the sample size regarding evaluation of cross‐protective efficacy. Globally, HPV‐31, HPV‐33 and HPV‐45 are among the most common types after HPV‐16 and HPV‐18, and are recognized as important causes of cervical cancer.^33^, ^34^, ^35^, ^36^ In China, HPV‐31, HPV‐33 and HPV‐45 together account for approximately 8% of cervical cancers.^5^ In addition, some epidemiology data show that HPV‐58 and HPV‐52 are the most prevalent types in Chinese women with cervical cancer after HPV‐16 and HPV‐18.^5^ In our study, vaccine efficacy against incident infection was 28.2% (95% CI: 6.0, 45.4) for HPV‐58 and 7.0% (95% CI: −8.7, 20.5) for HPV‐52 in the TVC‐E.

Vaccine efficacy against incident infection was higher than vaccine efficacy against 6MPI or 12MPI for some nonvaccine HPV types such as HPV‐45 and HPV‐58. This may be explained by a phenomenon known as masking by which infections with some nonvaccine types may have been missed by the SPF10‐LiPA assay used to genotype HPV types in this trial when HPV16 is present.^20^ Masking of nonvaccine type infections in women in the control group with multiple HPV infections may have led to a bias against the vaccine.^20^

To explore the potential overall effect of HPV vaccination, vaccine efficacy against CIN2+ irrespective of HPV DNA in the lesion was evaluated in the TVC‐naïve, comprising women who at baseline were DNA‐negative for all 14 oncogenic HPV types tested, seronegative for HPV‐16 and HPV‐18 and had negative cytology. The analysis of the overall effect of vaccination was conducted in the TVC‐naïve because these women had no evidence of HPV infection at baseline; the HPV vaccine is a prophylactic vaccine intended to be administered to girls before HPV infection can be acquired via sexual activity. However, all subjects in the current study were enrolled after their sexual debut. Therefore, it is possible that some women included in the TVC‐naïve cohort were not truly naïve and may have preexisting HPV infection that was not detected due to constraints of sampling or the laboratory assays used. In the present study, vaccine efficacy against CIN2+ irrespective of HPV DNA in the lesion was 33.5% (95% CI: −52.6, 72.1). This compares with vaccine efficacy of 64.9% (95% CI: 52.7, 74.2) against CIN2+ irrespective of HPV DNA in the lesion in the TVC‐naïve population of the PATRICIA trial.^12^

The observed difference of the point estimates in overall efficacy in HPV‐naïve women between the current study and PATRICIA may be explained by several factors related to sample size, study design and local HPV epidemiology. The present study enrolled approximately 6000 young women (approximately one third of the size of the PATRICIA trial sample) and was not powered to assess overall impact, which leads to a point estimate with a wide CI that includes the point estimate observed in PATRICIA.

Median age at enrolment was 23 years in the current study compared with 20 years in the PATRICIA trial. In addition, due to ethical and cultural considerations in China, only women who were already sexually active before the start of the study were enrolled in the trial, and the majority of women were already married with stable sexual relationships. Likely linked to these enrolment criteria, the incidence rates of CIN2+ irrespective of HPV in the control group of the TVC‐naïve population were very low in the current study (0.20 per 100 person‐years), more than four times lower compared with the PATRICIA trial (0.84 per 100 person‐years).^12^

Interestingly, the incidence rate of CIN2+ irrespective of HPV types in the current study is similar to that observed in the VIVIANE trial (0.19 in 100 follow‐up years) that enrolled women 25 years and older.^16^ In that study, a similarly low efficacy irrespective of type against CIN2+ was observed (6.6% [95% CI: −124.3, 61.4]) in the TVC‐naïve, suggesting an impact of age and/or sexual activity at time of vaccination, in spite of negative PCR results at baseline.

Another factor worth considering in the interpretation of overall vaccine impact is related to HPV prevalence and genotype distribution in different regions. While HPV‐16/18 are the most common oncogenic types accounting for cervical cancer in China,^5^, ^37^ it was observed in the current study that less than 40% of CIN2+ cases (6 out of 16, Figure 3) in the control group of the TVC‐naïve was associated with HPV‐16/18 only (4 cases) or associated with HPV‐16/18 and coinfected with a nonvaccine type (2 cases). HPV types 39, 52 and 58 were detected in 6 out of 16 CIN2+ lesions in the control group, and 6 out of 11 in the vaccine group of the TVC‐naïve. Cross‐protection has not been demonstrated consistently for these types.

We observed high and sustained anti‐HPV‐16 and anti‐HPV‐18 antibody levels in Chinese women, in line with previous long‐term studies.^16^, ^22^, ^38^ Long‐term protection against HPV infection is believed to be mediated via transudation of neutralizing antibodies induced by vaccination across the cervical epithelium to the site of HPV infection.^39^, ^40^, ^41^, ^42^ Mathematical modeling based on data from a previous study predicts that anti‐HPV‐16 and anti‐HPV‐18 antibody levels will remain above those associated with natural infection for at least 20 years postvaccination.^22^ We anticipate that the high antibody levels observed up to 72 months after first vaccination in our study will be sustained in the future, providing strong and long‐term protection against HPV infection in this population.

Serious AEs and AEs of interest (eg autoimmune disorders) occurred at a similar frequency in women receiving the AS04‐HPV‐16/18 vaccine or control, and at a comparable rate to data reported in global studies of women in a similar age group.^12^, ^14^, ^22^, ^23^ No fatal events or related SAEs occurred in the AS04‐HPV‐16/18 vaccine group. Most pregnancies resulted in a normal infant or were terminated electively. Few congenital anomalies observed in these women, who had had long gap between exposure to study vaccine and their last menstrual period prior to pregnancy (0.5 to 2.7 years). No pattern in the nature of congenital anomalies was identified and none was considered related to vaccination by the investigator. No safety signal concerning congenital anomalies has arisen during the AS04‐HPV‐16/18 vaccine trials, including the large PATRICIA trial and a pooled analysis of safety data which included over 10 000 pregnancies.^12^, ^23^ Moreover, the incidence of congenital anomalies in the study was low compared with the reported incidence in the general population of China.^43^, ^44^

Strengths and limitations of the study have been discussed previously.^24^, ^25^

**Figure 5:**
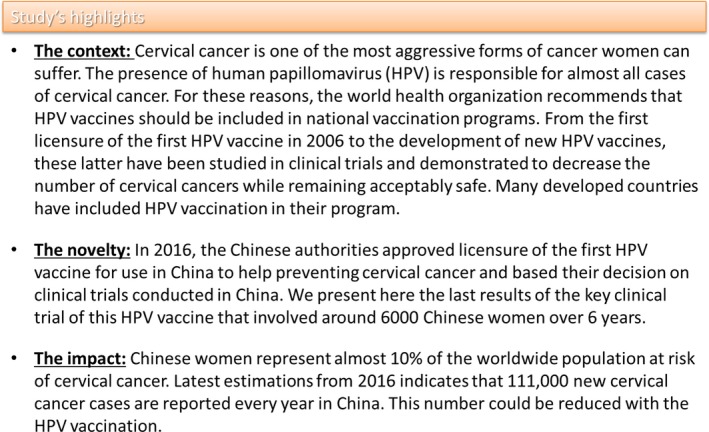
Study highlights

A lay language summary contextualizing the results and potential clinical research relevance and impact is displayed in Figure 5.

## 5 CONCLUSIONS

This long‐term efficacy study showed that the AS04‐HPV‐16/18 vaccine protects young Chinese women against infection, abnormal cytology and CIN lesions associated with HPV‐16/18 up to 72 months after first vaccination. In addition, data from this study on cross‐protection against HPV‐31, HPV‐33 and HPV‐45 are generally consistent with data from other trials of the same vaccine, including the large, multinational PATRICIA trial.^19^ Immunogenicity was high and sustained throughout the trial, and the vaccine had an acceptable safety profile. Overall, the results are consistent with those of large global trials of the AS04‐HPV‐16/18 vaccine.

## TRADEMARK STATEMENT

Cervarix is a trademark owned by or licensed to the GSK group of companies.

## ETHICS APPROVAL

The study protocol and informed consent were reviewed and approved by the ethics committees of the Center for Disease Control and Prevention (CDC) Jiangsu Province and the Cancer Foundation of China. Written informed consent was obtained from each participant prior to the performance of any study‐specific procedures.

## CONFLICT OF INTEREST

All authors have completed the Unified Competing Interest form at http://www.icmje.org/coi_disclosure.pdf and declare the following potential conflicts of interest: The institutions of Fang‐Hui Zhao, Shang‐Ying Hu, Ying Hong, Yue‐Mei Hu, Xun Zhang, Yi‐Ju Zhang, Qin‐Jing Pan, Wen‐Hua Zhang, Cheng‐Fu Zhang, Xiaoping Yang, Jia‐Xi Yu, Jiahong Zhu, Yejiang Zhu, Feng Chen, Qian Zhang, Hong Wang, Changrong Wang, Jun Bi, Shiyin Xue, Lingling Shen, Yan‐Shu Zhang, Feng‐Cai Zhu received grants/investigator fees from the GSK group of companies for the conduct of this study. Fang‐Hui Zhao, Feng‐Cai Zhu and Yue‐Mei Hu received support for travel to meetings related to the study from the GSK group of companies. Haiwen Tang, Naveen Karkada, Dan Bi and Frank Struyf are employees of the GSK group of companies. Haiwen Tang, Dan Bi, and Frank Struyf hold shares in the GSK group of companies. Pemmaraju Suryakiran and Yunkun He were employees of the GSK group of companies at the time the study was conducted.

## AUTHORS’ CONTRIBUTIONS

All authors participated in the design or implementation or analysis, and interpretation of the study; and the development of this manuscript. All authors had full access to the data and gave final approval before submission.

## Data Availability

Anonymized individual participant data and study documents can be requested for further research from https://www.clinicalstudydatarequest.com
