# Supplementary material for: Efficacy, immunogenicity and safety of the AS04‐HPV‐16/18 vaccine in Chinese women aged 18‐25 years: End‐of‐study results from a phase II/III, randomised, controlled trial
Source: Cancer Med. 2019 Jul 15;8(14):6195–211. doi: 10.1002/cam4.2399 (PMC6797633; doi:10.1002/cam4.2399)
Supplement: Supplementary file 1 [file CAM4-8-6195-s001.docx]

# Supporting Information

**Efficacy, immunogenicity and safety of the AS04-HPV-16/18 vaccine in Chinese women aged 18–25 years: end-of-study results from a phase II/III, randomised, controlled trial**

Feng-Cai Zhu, Shang-Ying Hu, Ying Hong, Yue-Mei Hu, Xun Zhang, Yi-Ju Zhang, Qin-Jing Pan, Wen-Hua Zhang, Fang-Hui Zhao, Cheng-Fu Zhang, Xiaoping Yang, Jia-Xi Yu, Jiahong Zhu, Yejiang Zhu, Feng Chen, Qian Zhang, Hong Wang, Changrong Wang, Jun Bi, Shiyin Xue, Lingling Shen, Yan-Shu Zhang, Yunkun He, Haiwen Tang, Naveen Karkada, Pemmaraju Suryakiran, Dan Bi, Frank Struyf

Cancer Medicine

^†^Correspondence to:

Professor Fang-Hui Zhao

National Cancer Center, Cancer Institute and Hospital, Chinese Academy of Medical Sciences and Peking Union Medical College, 17 South Panjiayuan Lane, P.O. Box 2258, Beijing 100021, China

E-mail: [zhaofangh@cicams.ac.cn](mailto:zhaofangh@cicams.ac.cn)

Content of supporting information:

Supplementary Table 1. Vaccine efficacy against CIN1+ and CIN2+ associated with non-vaccine HPV types

Supplementary Table 1. Vaccine efficacy against CIN1+ and CIN2+ associated with non-vaccine HPV types

|  | **ATP-E, women DNA-negative for HPV type considered at baseline and Month 6, regardless of HPV-16/18 serostatus** | | | | | **TVC-E, women DNA-negative for HPV type considered at baseline, regardless of HPV-16/18 serostatus** | | | | |
| --- | --- | --- | --- | --- | --- | --- | --- | --- | --- | --- |
|  | **Vaccine** | | **Control** | | **Vaccine efficacy, % (95% CI)** | **Vaccine** | | **Control** | | **Vaccine efficacy, % (95% CI)** |
|  | **N** | **n** | **N** | **n** |  | **N** | **n** | **N** | **n** |  |
| **CIN1+** | | | | | | | | | | |
| **HPV-31** | 2773 | 0 | 2769 | 6 | 100 (14.9, 100) | 2830 | 2 | 2841 | 7 | 71.2 (-51.3, 97.1) |
| **HPV-33** | 2766 | 5 | 2770 | 3 | -67.6 (-979.4, 67.4) | 2825 | 8 | 2840 | 3 | -169.5 (-1477.4, 35.3) |
| **HPV-35** | 2792 | 2 | 2790 | 3 | 33.1 (-484.5, 94.4) | 2842 | 2 | 2852 | 5 | 59.7 (-146.1, 96.2) |
| **HPV-39** | 2744 | 7 | 2759 | 1 | -608.1 (-31813.4, 9.0) | 2811 | 8 | 2833 | 3 | -170.3 (-1481.9, 35.1) |
| **HPV-45** | 2780 | 3 | 2788 | 3 | -0.8 (-652.6, 86.5) | 2838 | 4 | 2852 | 3 | -34.7 (-819.2, 77.2) |
| **HPV-51** | 2740 | 3 | 2742 | 9 | 66.5 (-34.1, 94.2) | 2816 | 4 | 2812 | 11 | 63.6 (-22.9, 91.5) |
| **HPV-52** | 2652 | 11 | 2651 | 12 | 7.5 (-129.0, 63.0) | 2752 | 15 | 2750 | 18 | 16.2 (-76.0, 60.7) |
| **HPV-56** | 2769 | 2 | 2766 | 4 | 49.9 (-249.8, 95.5) | 2826 | 2 | 2831 | 4 | 49.7 (-251.1, 95.5) |
| **HPV-58** | 2758 | 6 | 2755 | 9 | 33.0 (-110.7, 80.4) | 2818 | 9 | 2823 | 12 | 24.5 (-95.3, 71.9) |
| **HPV-59** | 2800 | 1 | 2783 | 0 | N/E | 2851 | 2 | 2848 | 3 | 33.1 (-483.9, 94.4) |
| **HPV-66** | 2766 | 6 | 2751 | 8 | 25.0 (-146.5, 78.6) | 2830 | 7 | 2820 | 8 | 12.3 (-176.7, 72.9) |
| **HPV-68** | 2761 | 3 | 2771 | 3 | -0.6 (-651.4, 86.5) | 2828 | 3 | 2840 | 4 | 24.4 (-346.7, 88.9) |
| **HPV-31/33/45** | 2809 | 7 | 2810 | 11 | 36.1 (-80.5, 79.0) | 2857 | 12 | 2864 | 12 | -0.8 (-145.2, 58.6) |
| **HRW-HPV** | 2809 | 42 | 2811 | 53 | 20.5 (-21.5, 48.3) | 2857 | 54 | 2864 | 67 | 19.0 (-17.7, 44.4) |
| **HR-HPV** | 2809 | 42 | 2811 | 64 | 34.3 (1.5, 56.6) | 2857 | 56 | 2864 | 78 | 27.9 (-2.9, 49.8) |
| **CIN2+** | | | | | | | | | | |
| **HPV-31** | 2773 | 0 | 2769 | 4 | 100 (-51.9, 100) | 2830 | 1 | 2841 | 5 | 79.8 (-80.2, 99.6) |
| **HPV-33** | 2766 | 2 | 2770 | 3 | 33.0 (-485.1, 94.4) | 2825 | 5 | 2840 | 3 | -68.4 (-984.5, 67.2) |
| **HPV-35** | 2792 | 1 | 2790 | 2 | 49.8 (-864.5, 99.2) | 2842 | 1 | 2852 | 3 | 66.4 (-318.3, 99.4) |
| **HPV-39** | 2744 | 2 | 2759 | 0 | N/E | 2811 | 3 | 2833 | 1 | -204.0 (-15860.5, 75.6) |
| **HPV-45** | 2780 | 0 | 2788 | 2 | 100 (-436.5, 100) | 2838 | 1 | 2852 | 2 | 49.5 (-869.5, 99.1) |
| **HPV-51** | 2740 | 1 | 2742 | 0 | N/E | 2816 | 1 | 2812 | 1 | -0.3 (-7775.1, 98.7) |
| **HPV-52** | 2652 | 2 | 2651 | 2 | -0.9 (-1292.1, 92.7) | 2752 | 3 | 2750 | 5 | 39.7 (-210.1, 90.6) |
| **HPV-56** | 2769 | 0 | 2766 | 1 | 100 (-3811.4, 100) | 2826 | 0 | 2831 | 1 | 100 (-3826.1, 100) |
| **HPV-58** | 2758 | 5 | 2755 | 5 | -0.5 (-336.7, 76.9) | 2818 | 7 | 2823 | 6 | -17.6 (-323.5, 66.2) |
| **HPV-59** | 2800 | 0 | 2783 | 0 | N/E | 2851 | 0 | 2848 | 0 | N/E |
| **HPV-66** | 2766 | 2 | 2751 | 1 | -100 (-11696.8, 89.6) | 2830 | 2 | 2820 | 1 | -100.4 (-11719.8, 89.6) |
| **HPV-68** | 2761 | 0 | 2771 | 0 | N/E | 2828 | 0 | 2840 | 0 | N/E |
| **HPV-31/33/45** | 2809 | 3 | 2811 | 18 | 74.9 (-25.8, 97.4) | 2857 | 6 | 2864 | 9 | 32.9 (-111.2, 80.3) |
| **HRW-HPV** | 2809 | 13 | 2811 | 17 | 23.2 (-68.0, 65.7) | 2857 | 20 | 2864 | 23 | 12.5 (-66.7, 54.4) |
| **HR-HPV** | 2809 | 13 | 2811 | 26 | 49.8 (-1.2, 76.3) | 2857 | 21 | 2864 | 33 | 36.0 (-13.9, 64.8) |

ATP-E: according to protocol cohort for efficacy; CIN1(2)+: cervical intraepithelial neoplasia CIN grade 1 (2) or worse; CI: confidence interval; HPV: human papillomavirus; HR-HPV: any oncogenic HPV type including HPV-16 or HPV-18; HRW-HPV: any oncogenic HPV type except HPV-16 and HPV-18; N: number of women in analysis; n: number of cases; N/E: not able to estimate; TVC-E: total vaccinated cohort for efficacy. For combined types, women were DNA-negative at baseline (ATP-E and TVC-E) and Month 6 (ATP-E) for at least one of the HPV types considered in the analysis. Women could be infected with multiple HPV types. Therefore, the number of cases for combined types (HPV-31/33/45, HRW-HPV and HR-HPV) might not equal the sum of the cases for each individual type included in the composite.
